# Sulforaphane Ameliorates Nonalcoholic Fatty Liver Disease Induced by High-Fat and High-Fructose Diet via LPS/TLR4 in the Gut–Liver Axis

**DOI:** 10.3390/nu15030743

**Published:** 2023-02-01

**Authors:** Ye Xu, Xianghui Huang, Bingxin Huangfu, Yanzhou Hu, Jia Xu, Ruxin Gao, Kunlun Huang, Xiaoyun He

**Affiliations:** 1 Key Laboratory of Precision Nutrition and Food Quality, Key Laboratory of Functional Dairy, Ministry of Education, College of Food Science and Nutritional Engineering, China Agricultural University, Beijing 100083, China; 2 Henan Shuanghui Investment and Development Co., Ltd., Luohe 462000, China; 3 Key Laboratory of Safety Assessment of Genetically Modified Organism (Food Safety), The Ministry of Agriculture and Rural Affairs of the P.R. China, Beijing 100083, China

**Keywords:** non-alcoholic fatty liver disease, sulforaphane, gut–liver axis, LPS/TLR4, inflammation

## Abstract

The gut–liver axis has emerged as a key player in the progression of non-alcoholic fatty liver disease (NAFLD). Sulforaphane (SFN) is a bioactive compound found in cruciferous vegetables; however, it has not been reported whether SFN improves NAFLD via the gut–liver axis. C57BL/6 mice were fed a high-fat and high-fructose (HFHFr) diet, with or without SFN gavage at doses of 15 and 30 mg·kg^−1^ body weight for 12 weeks. The results showed that SFN reduced weight gain, hepatic inflammation, and steatosis in HFHFr mice. SFN altered the composition of gut microbes. Moreover, SFN enhanced the intestinal tight junction protein ZO-1, reduced serum LPS, and inhibited LPS/TLR4 and ERS pathways to reduce intestinal inflammation. As a result, SFN protected the intestinal integrity and declined the gut-derived LPS translocations to the liver in HFHFr diet-induced mice. SFN decreased the liver LPS levels and inhibited the LPS/TLR4 pathway activations, thus inhibiting the pro-inflammatory cytokines. Notably, Spearman correlation analysis showed that the protective effect of SFN on intestinal barrier integrity and its anti-inflammatory effect on the liver was associated with improved intestinal dysbiosis. Above all, dietary intervention with SFN attenuates NAFLD through the gut–liver axis.

## 1. Introduction

Non-alcoholic fatty liver disease (NAFLD) is one of the most common liver diseases worldwide. It is characterized by excessive hepatic steatosis, lobular inflammation, and hepatocyte ballooning changes. Moreover, NAFLD may progress to non-alcoholic steatohepatitis (NASH) and cirrhosis, potentially leading to hepatic carcinoma [1]. NAFLD pathogenesis may be linked to abnormal glucose and lipid metabolism, inflammation, endoplasmic reticulum stress (ERS), oxidative stress, and imbalanced gut microorganisms, according to existing research [2,3]. However, safe and effective NAFLD therapies have yet to be discovered. Therefore, uncovering the pathogenesis of NAFLD and developing prevention and treatment strategies for NAFLD remains challenging for fatty liver research.

The hepatic portal system anatomically connects the liver and gut and enables gut microbiota and their metabolites to be involved in the pathogenesis of NAFLD. Hence, the gut–liver axis is increasingly recognized as a critical factor in NAFLD [4]. Existing studies have observed intestinal flora disturbances and increased intestinal permeability in NAFLD animal models and NAFLD patients [3,5,6,7]. The disturbance of gut microbiota may increase serum lipopolysaccharide (LPS), the Gram-negative bacteria’s main outer membrane compound; LPS may damage the intestinal barrier function and increase intestinal permeability [8]. A clinical study has shown that in patients with NAFLD, gut permeability caused by disruption of intercellular tight junctions in the intestine, is correlated with the severity of steatosis [9]. Likewise, animal studies found that the intestinal mucosal protectant sodium butyrate reduced liver pathology in NAFLD mice, indicating that intestinal barrier integrity helps with NAFLD [10]. The tight junction proteins regulate gut permeability by occupying paracellular positions between adjacent enterocytes, allowing the passage of ions and small molecules, and preventing the transformations of gut microorganisms to the liver [11]. The gut barrier integrity damage due to gut microbiota disturbance is found to be a prerequisite for NASH development [12]. Meanwhile, the intestinal epithelial cells’ unfolded protein response (UPR), and impaired endoplasmic reticulum function damages were reported in obese individuals [13]. Intestinal inflammation is related to ERS. The increased expression of ERS-related genes GPR78 and CHOP protein is accompanied by the up-regulation of intestinal inflammatory genes such as IL-1β and TNF-α [14]. Therefore, gut microorganism disorders and ERS may disrupt intestinal integrity and enables gut bacteria-produced LPS to enter the portal vein through the blood circulation, thereby promoting inflammatory responses in the liver.

Several studies have shown that LPS and its downstream pathways significantly impact NAFLD-induced liver inflammation [15,16,17]. LPS can be recognized by the pattern recognition receptor, Toll-like receptor 4 (TLR4), and trigger a lower-level immune response [18]. Once TLR4 is activated, the essential adaptor protein myeloid differentiation primary response 88 (MyD88) is synchronously activated, activating nuclear factor-κB (NF-κB) and provoking inflammation, contributing to releasing inflammatory cytokines [19]. Thus, TLR4/NF-κB inflammatory pathway activations, mediated by gut-derived LPS, may be crucial in the occurrence and progression of NASH.

Sulforaphane (SFN) is a natural isothiocyanate compound found in cruciferous vegetables with robust anti-inflammatory and anticancer properties [20]. Studies have shown that glucoraphanin, a precursor of SFN, reduces obesity and insulin resistance in mice [21]. SFN is the most potent natural inducer of nuclear factor erythroid 2-related factor 2 (NRF2) and protects mitochondrial functions, thereby inhibiting the development of NASH [22]. SFN also improves lipid metabolism disorders in NAFLD mice by up-regulating the fibroblast growth factor (FGF) 21/FGFR1 pathway [23]. Recent studies indicate that SFN showed multiple protective effects on intestinal inflammation [24]. However, our hypothesis on the SFN’s improvement effect against NAFLD by enhancing the gut barrier, modulating gut microbiota, and LPS-mediated gut–liver axis remains to be elucidated.

Hence, this study used a high-fat and high-fructose diet to construct a NAFLD model. The purpose of this study is to verify SFN’s impact on gut microbes, intestinal barrier integrity, and inflammation in NAFLD mice. The potential mechanism of SFN against NAFLD is thought to be a LPS-mediated TLR4/NF-κB signaling pathway.

## 2. Materials and Methods

### 2.1. Regents

SFN used in the animal was purchased from Aladdin (S111997, HPLC ≥ 95%, Shanghai, China). Fructose (C6H12O6, HPLC ≥ 99%) was purchased from Xiwang Sugar (Shangdong, China). A high-fat diet (HFD) (60% of energy derived from fat) was purchased from Research Diets (New Brunswick, NJ, USA). The maintenance diet was purchased from Keao Xieli (Beijing, China).

### 2.2. Animals and Treatments

**Figure 1:**
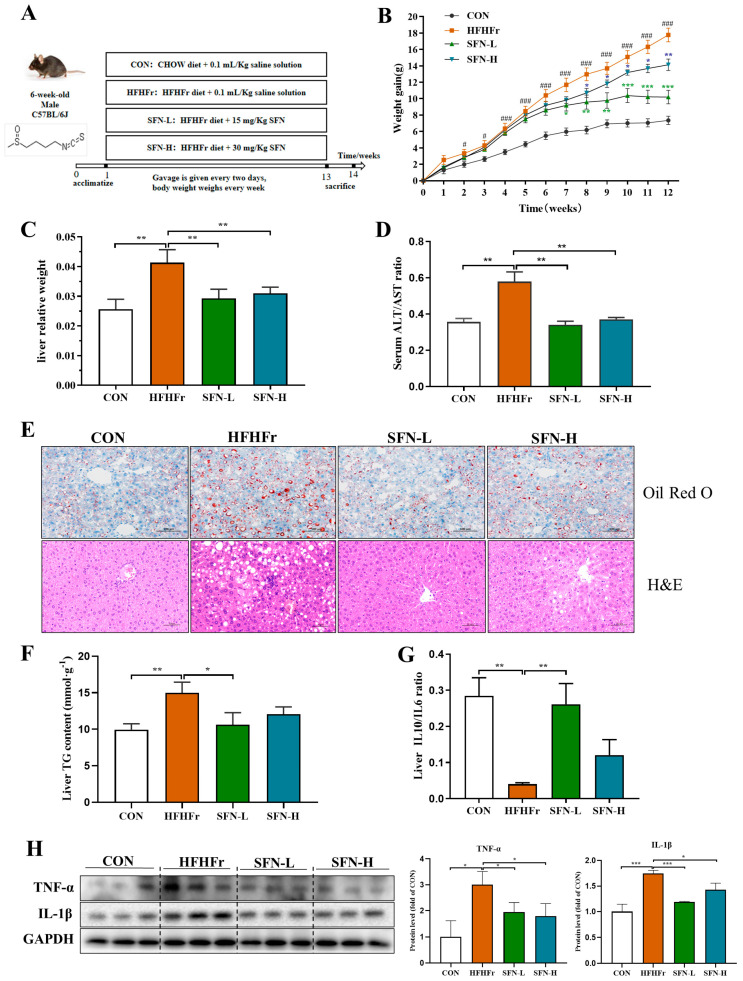
SFN inhibits body weight gain and alleviates hepatic steatosis in HFHFr-fed mice. (**A**) The flow chart of animal treatments. (**B**) Body weight gain curve (*n* = 8). (**C**) Liver weight (*n* = 8). (**D**) Serum ALT/AST ratio (*n* = 6). (**E**) Representative images of liver tissues stained by Oil Red O staining and H&E staining in mice (*n* = 6). The scale bar of Oil Red O staining is 100 µm and the scale bar of H&E staining is 50 µm. (**F**) Content of TG in livers (*n* = 6). (**G**) The ratio of IL-10/IL-6 in the liver was measured by ELISA (*n* = 6). (**H**) The protein expression of TNF–α, and IL-1β in the liver was detected by Western blot (*n* = 3). Data are shown as mean ± SEM. * *p* < 0.05, ** *p* < 0.01, *** *p* < 0.001 compared with HFHFr, ^#^ *p* < 0.05, ^###^ *p* < 0.001 compared with CON.

Six-week-old male C57BL/6J mice were purchased from Beijing Charles River Laboratories (Beijing, China) and housed in an SPF animal house (22 ± 2 °C, 40~70% relative humidity, 12 h light/12 h dark cycles). The animal experiment design was shown in Figure 1A. After one-week acclimatization, they were randomly divided into four groups (8 mice per group): (1) mice fed the control diet (pure water and maintenance diet) as a control (CON) group; (2) mice fed the high fructose (30% (W/V) fructose water and HFD as the HFHFr group; (3) mice fed the HFHFr diet supplemented with a low dose of SFN (15 mg/kg) as the SFN-L group; and (4) mice fed the HFHFr diet supplemented with a high dose of SFN (30 mg/kg) as the SFN-H group. SFN was given to mice every two days by gavage in a sterile saline solution. The CON group and HFHFr group were given sterile saline by gavage at a dose of 0.1 mL·10 g^−1^·body weight every two days to minimize the effects of the gavage procedure. The experiment lasted for 12 weeks during which the body weight of each mouse was monitored weekly. Fecal samples from each mouse were collected during the last week before the animals were euthanized. After 12 weeks, all animals were fasted for 12 h and then sacrificed. The organs were dissected immediately. The livers were weighed. Some of the liver and colon tissues of mice were fixed with 4% paraformaldehyde, and the rest were used for molecular and biochemical tests.

All the experimental procedures were performed according to the Guide for the Care and Use of Laboratory Animals of the National Institutes of Health. The study was approved under the regulations of the Committee on the Ethics of Animal Experiments of China Agricultural University, Beijing (approval ID: Aw10401202-4-4).

### 2.3. Dose Information

According to previous reports, SFN was orally administered at a dose of 10, 25, and 50 mg·kg^−1^ body weight in mice [25,26,27]. In our study, SFN doses were selected based on previous research and our preliminary experiments. The equivalent low and high SFN doses for humans are 1.648 and 3.298 mg·kg−1 body weight, respectively, based on the Meeh-Rubner equation [28,29].

### 2.4. Determination of Biochemical Indications in Mice

Serums of mice were collected, and a biochemistry analyzer (98640000, Indiko™ Plus Clinical Chemistry Analyzer, Thermo Fisher Scientific, Waltham, MA, USA) was used to determine the aspartate aminotransferase (AST), and alanine aminotransferase (ALT).

### 2.5. Cytokine Enzyme-Linked Immunosorbent Assay (ELISA)

The contents of liver IL-6 and IL-10 were determined by enzyme-linked immune sorbent assay (ELISA) kits (R&D Systems, Minneapolis, MN, USA) by the manufacturer’s protocols. The final results of IL-6 and IL-10 were normalized to total protein concentration.

### 2.6. Determination of Lipopolysaccharide in the Liver and Serum of Mice

The quantification of liver and serum LPS was performed following the instructions of the commercial kits (CUSABIO, Wuhan, China). The final result of the liver LPS was normalized to total protein concentration.

### 2.7. Histological Analysis

Briefly, frozen liver sections (10 μm) were stained with 0.5% Oil Red O (Sigma-Aldrich, St. Louis, MO, USA) for 15 min and then washed. Three fields from three sections of each mouse were viewed under a Leica microscope, and digital photographs were captured.

The liver and colon tissues were fixed in 10% (*v/v*) formalin overnight before being subjected to gradient ethanol dehydration. Then, the tissues were embedded, sliced, and stained with hematoxylin and eosin (H&E). The sections were scanned by 3DHISTECH Pannoramic SCAN (Budapest, Hungary). Microphotographs were taken by CaseViewer 2.4.

For immunohistochemistry (IHC), the sections of the colon were incubated in primary antibodies including ZO-1(Abcam, Waltham, MA, USA), TLR4, MyD88, and NF-κB (Proteintech Group, Inc. (Wuhan, China)). Secondary antibodies labeled with horseradish peroxidase were used. Detection was conducted using a horseradish peroxidase–based commercial detection system, disclosure with diaminobenzidine chromogen, and nuclear counterstaining with hematoxylin. The sections were observed under an Olympus BX60 microscope (Tokyo, Japan). The intensity of brown staining was analyzed using Image Pro Plus (version 6.0).

### 2.8. Real-Time Quantitative PCR

RNA was reverse-transcribed into cDNA with a One-Step gDNA Removal and cDNA Synthesis SuperMix (AT311, TransGen Biotech, Beijing, China). Real-time quantitative PCR (qPCR) was performed with a SuperReal PreMix Plus (FP 205-03, TIANGEN Biotech, Beijing, China) by using a real-time PCR system (C1000, Bio-Rad, Hercules, CA, USA). Gene expression levels were normalized to β-actin. Primer sequences are listed in Supplementary Table S1.

### 2.9. Western Blot Analysis

For Western blot analysis, standard SDS-PAGE blotting methods were used. Primary antibodies used in Western blot are as follows: GAPDH (Beyotime, Beijing, China); TLR4, MyD88, and NF-κB (Proteintech Group, Inc., Wuhan, China); TNF-α and IL-1β (Abcam, Waltham, MA, USA). Chemiluminescence was visualized using an imaging system (330037, Clinx Science Instruments Co. Ltd., Shanghai, China).

### 2.10. Gut Microbiota Analysis

Microbial genomic DNA was extracted using a previously described method [30]. The bacterial V3 + V4 regions of the 16S rRNA were amplified and sequenced on a Nova-Seq platform (Illumina, San Diego, CA, USA). The raw data was filtered and analyzed by QIIME (v1.9.1). UPARSE v7.0.1001 was used to cluster the OTUs at an identity threshold of 97%. Nonmetric multidimensional scaling (NMDS) plots were analyzed using PAST v2.17 based on Bray–Curtis distance. LEfSe analysis was performed to find the differentially abundant biomarker between the experimental groups. Correlations between the experimental result parameters and the microbiota profiles were analyzed by Spearman correlational analysis.

### 2.11. Statistical Analysis

The results are presented as means ± SEMs. All data differences were analyzed by one-way ANOVA (Tukey’s multiple comparison tests) on GraphPad Prism (version 8.0), which was considered significant at *p* < 0.05.

## 3. Result

### 3.1. SFN Improves Weight Gain and Hepatic Steatosis in HFHFr-Diet Mice

As shown in Figure 1A, NAFLD mice models were generated with a 12-weeks HFHFr-diet. SFN-L treatment slowed the mice’s weight gains from the eighth week and kept their body weight steady in the last three weeks (Figure 1B). Likewise, the weight gain of the SFN-H-treated mice decreased. Although the decrease levels were weaker than in the SFN-L group, no statistical differences were observed between the two groups. These data indicated that SFN significantly inhibited the weight gain of HFHFr mice. Additionally, the liver weight of SFN-treated mice was lower than the HFHFr group (Figure 1C). Serum ALT/AST ratio levels commonly used in liver injury assessments [31] were significantly decreased in the SFN-treated mice (Figure 1D).

Oil Red O staining revealed that (Figure 1E) the number and size of liver lipid droplets increased significantly in HFHFr mice and were improved by SFN-L or SFN-H treatments. The H&E staining showed clear and complete hepatic lobules in CON mice, with no evidence of steatosis and focal inflammatory infiltrations. In contrast to the CON group, the hepatic lobules of HFHFr mice were less complicated, with a variety of vacuolar lipid droplets, hepatocyte disorders, and severe inflammatory infiltration. The SFN supplement improved the mice’s vacuole numbers and inflammatory cell infiltrations (Figure 1E). Moreover, the intervention of low-dose SFN significantly reduced the content of TG in the liver (Figure 1F). We assessed the regulatory effect of SFN on hepatic pro- and anti-inflammatory factors by using Il-10/Il-6. The results showed that SFN treatment reversed the decreased IL10/IL6 ratio, suggesting that it alleviated inflammation in the liver (Figure 1G). Additionally, pro-inflammatory factors TNF-α and IL-1β were decreased in the SFN group compared with the HFHFr group (Figure 1H). Although the SFN-L treatment showed more robust anti-inflammatory effects than the SFN-H treatment, there was no statistical difference between the two groups. These findings suggest that SFN intervention improves HFHFr diet-induced hepatic lipid accumulation, hepatic steatosis, and inflammatory infiltration in NAFLD mice.

### 3.2. SFN Improves Gut Dysbiosis Induced by the HFHFr Diet

**Figure 2:**
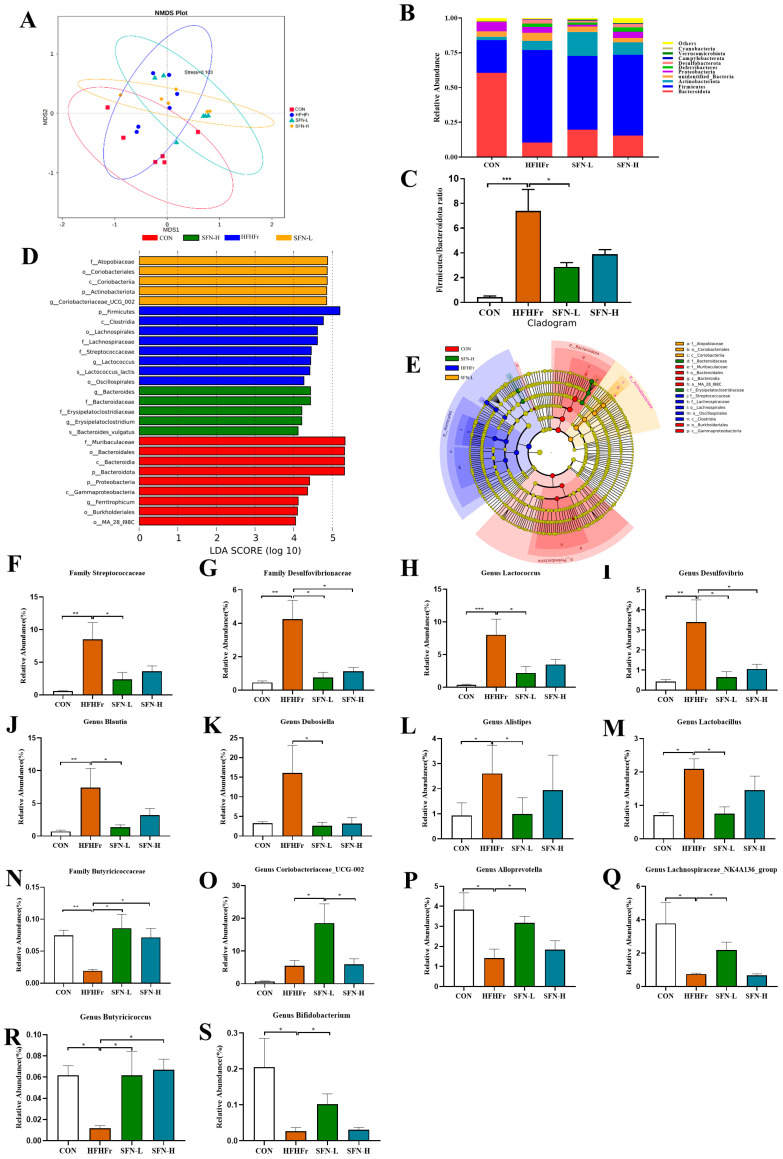
The intervention of SFN improves gut dysbiosis induced by the HFHFr diet. (**A**) Nonmetric multidimensional scaling (NMDS) plot based on the Bray–Curtis distance. (**B**) The relative abundance of the top ten abundant bacteria at the phylum level. (**C**) The ratio of Firmicutes/Bacteroidota. (**D**) Linear discriminate analysis effect size (LEfSe) analysis of the dominant biomarker taxa among the four groups. The threshold of the logarithmic score of LDA analysis was 4.0. (**E**) Taxonomic cladogram obtained from LEfSe analysis by comparing 4 groups. (**F**–**S**) Relative abundances of 14 significantly altered bacterial genera or families: (**F**) Streptococcaceae, (**G**) Desulfovibrionaceae, (**H**) *Lactococcus*, (**I**) *Desulfovibrio*, (**J**) *Blautia*, (**K**) *Dubosiella*, (**L**) *Alistipes*, (**M**) *Lactobacillus*, (**N**) Butyricicoccaceae, (**O**) *Coriobacteriaceae UCG_002*, (**P**) *Alloprevotella*, (**Q**) *Lachnospiraceae_NK4A136_group*, (**R**) *Butyricicoccus*, and (**S**) *Bifidobacterium*. Data are presented as mean ± SEM (*n* = 6). * *p* < 0.05, ** *p* < 0.01, *** *p* < 0.001 compared with HFHFr.

An increasing number of studies have reported gut microorganism disorders in NAFLD individuals, indicating their predominant role in NAFLD development [32,33]. We used 16S RNA gene sequencing on mice feces to investigate SFN’s role in NAFLD by regulating gut microbiota. NMDS analysis is a nonlinear model based on the Bray–Curtis distance. An NMDS stress of less than 0.2 means that this model accurately reflects the sample differences. Figure 2A shows the differences among samples from the experimental groups. Despite some overlaps, distinctive clustering between the HFHFr and SFN-treated mice can be observed, revealing specific differences in gut microbiota compositions after SFN intervention. We found that the differential bacteria at the phylum level were mainly Bacteroidota, Firmicutes, unidentified_Bacteria, Proteobacteria, Deferribacteres, Desulfobacterota, Campylobacterota, Verrucomicrobiota, and Cyanobacteria (Figure 2B). We found that HFHFr-diet increased the Firmicutes/Bacteroidota ratio (F/B ratio) and significantly altered the composition of gut microbiota (Figure 2C). SFN reduced the F/B ratio, and a statistical difference was observed between the HFHFr group and the SFN-L group.

A LEfSe (LDA > 4) analysis was performed to identify the significantly regulated specific phylotypes among the groups. Overall, 27 OTUs with significant abundance changes were screened as key phylotypes (Figure 2D). Based on the LDA score, we found that the HFHFr treatment enriched the phylum Firmicutes, such as the family Lachnospiraceae, family Streptococcacea, and genus *Lactococcus*. The SFN-L treatment, however, enriched the family Atopobiaceae, phylum Actinobacteriota, and genus *Coriobacteriaceae_UCG_002* (Figure 2D). Different levels of marker taxa in experimental groups obtained from LEfSe were also supported by branch graph analysis (Figure 2E).

Furthermore, we analyzed the dominant bacteria under SFN treatment. We noticed that HFHFr-diet increased the relative abundance of the family Streptococcaceae, Desulfovibrionaceae, genera *Lactococcus*, *Desulfovibrio*, *Blautia*, *Dubosiella*, *Alistipe*, and *Lactobacillus*, which are significantly declined by SFN treatment, especially in the low-dose treated mice (Figure 2F–M). Existing studies have reported that the family Streptococcaceae is enriched in colonic inflammation mice [34]. The family Desulfovibrionaceae and the genus *Desulfovibrio* can produce LPS, resulting in increased LPS in the blood [35]. *Lactococcus* and *Lactobacillus* are positively related to body weight and liver weight [36]. Additionally, *Blautia*, *Dubosiella*, and *Alistipe* were raised in HFD-diet mice. These findings indicated that SFN reduces harmful gut bacteria. In contrast to the HFHFr group, SFN-L treatment enriched the family Butyricicoccaceae, genera *Coriobacteriaceae_UCG-002*, *Alloprevotella*, *Lachnospiraceae_NK4A136_group*, *Butyricicoccus* and *Bifidobacterium* (Figure 2N–S). Notably, the SFN-L supplement significantly increased the relative abundance of *Coriobacteriaceae_UCG-002* compared with the other three groups (Figure 2O). Butyricicoccaceae, *Alloprevotella*, *Lachnospiraceae_NK4A136_group*, *Butyricicoccus*, and Bifidobacterium were decreased in the HFHFr group. These short-chain fatty acid-producing bacteria improve gut permeability and protect the intestinal barrier [37,38,39]. The relative abundance of these bacteria increased significantly after SFN intervention, and the lower dose treatment showed a better effect. Overall, these results confirm that SFN intervention improves the gut microbiota of the HFHFr-diet mice.

### 3.3. SFN Ameliorates Intestinal Histopathology and Improves Intestinal Permeability in HFHFr Mice

**Figure 3:**
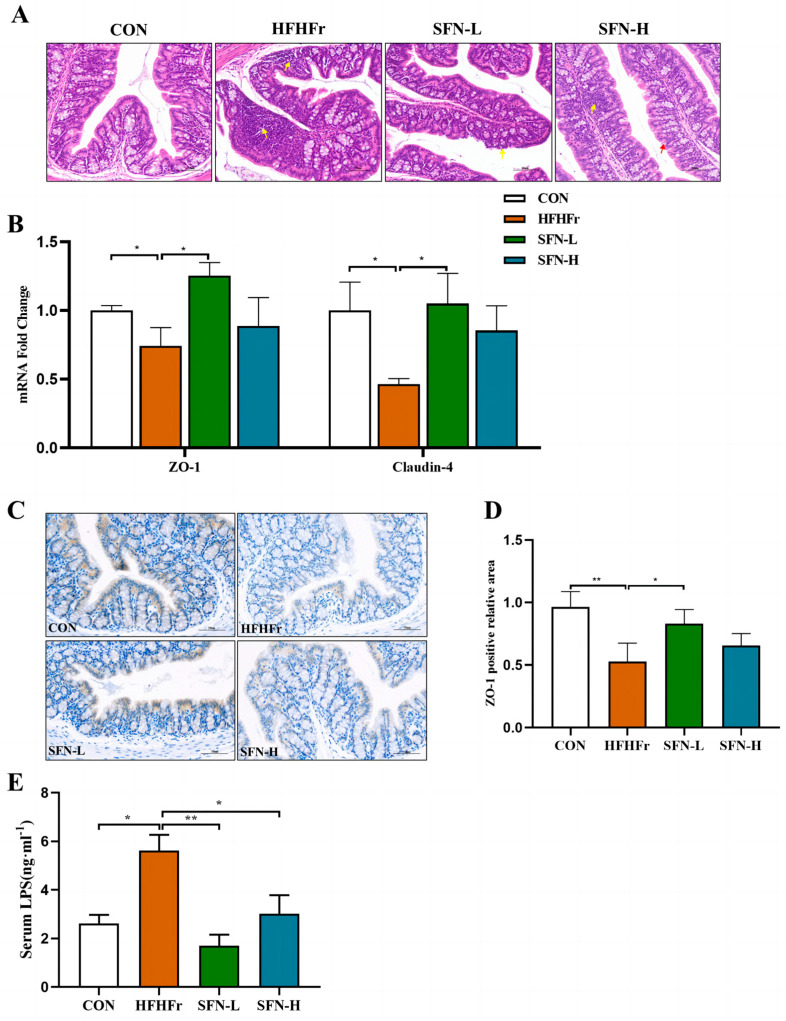
SFN ameliorates intestinal histopathology and improves intestinal permeability in HFHFr mice. (**A**) Representative images of colon tissues stained by H&E staining in mice (*n* = 6). (**B**) Relative mRNA levels analysis of ZO-1 and Claudin-4 in colon tissues (*n* = 6). (**C**) Representative images of immunohistochemical staining of ZO-1 in colon samples (*n* = 6). The scale bar is 50 µm. (**D**) Quantification of immunohistochemical staining for ZO-1 (*n* = 6). (**E**) Serum LPS concentration of each group (*n* = 6). Data are shown as mean ± SEM. * *p* < 0.05, ** *p* < 0.01 compared with HFHFr.

The disruption of intercellular tight junctions contributes to increased intestinal permeability and nerve function in the pathogenesis of hepatic steatosis. So, we next assessed whether SFN intervention ameliorated intestinal damage in HFHFr mice. As displayed in Figure 3A, the intestinal tissue structure of the mice in the HFHFr group was severely abnormal, the local crypt structure in the mucosal layer of the colon tissue disappeared, and a large number of inflammatory cells was infiltrated by H&E staining, while these phenomena were improved in the SFN group. This result indicated that SFN intervention improved intestinal histomorphology and ameliorated intestinal damage.

Since tight junctions maintain the intestinal epithelial barrier, defects that may lead to intestinal damage, we assessed the integrity of the intestinal barrier. The transcript levels of intestinal epithelial proteins ZO-1 and Claudin-4 were decreased in the HFHFr group, while the mRNA levels were significantly upregulated in the SFN-L group (Figure 3B). We further performed IHC to evaluate the expression of the intestinal tight junction protein ZO-1, and an elevated level of ZO-1 protein expression was observed in SFN groups compared with the HFHFr group (Figure 3C,D). Additionally, impaired intestinal barrier function and intestinal flora disruption can result in increased production of LPS, a biomarker of intestinal permeability. As shown in Figure 3E, serum LPS in the HFHFr group was significantly higher, in contrast, SFN significantly reduced the level of serum LPS. These results suggest that SFN can restore the integrity of the intestinal mucosal barrier disrupted and lower intestinal permeability by upregulating tight junction proteins in the HFHFr diet.

### 3.4. Intervention of SFN Alleviates Intestinal Inflammation in HHFFr Mice by Inhibiting TLR4/NF-κB and ERS Pathway

**Figure 4:**
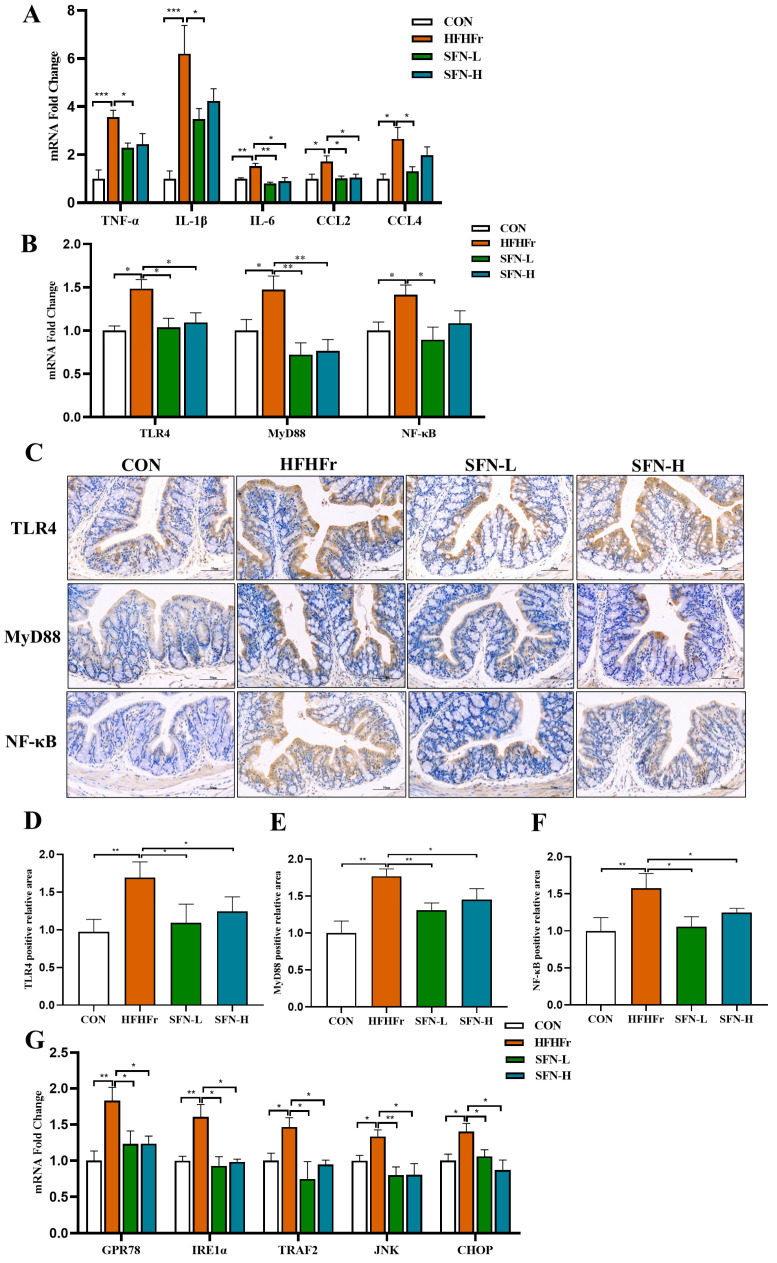
The intervention of SFN alleviates intestinal inflammation in HFHFr mice by inhibiting the TLR4 /NF-κB pathway and inhibiting endoplasmic reticulum stress. (**A**) The relative mRNA expression of the proinflammatory genes TNFα, IL-1β, and IL-6 and the chemokines CCL2 and CCL4 in the colon (*n* = 6). (**B**) Relative mRNA levels analysis of TLR4, MyD88, and NF-κB in the colon (*n* = 6). (**C**) Representative images of immunohistochemical staining of TLR4, MyD88, and NF-κB in colon samples (*n* = 6). The scale bar is 50 µm. (**D**–**F**) Quantification of immunohistochemical staining for TLR4, MyD88, and NF-κB (*n* = 6). (**G**) The relative mRNA expression of endoplasmic reticulum stress-related genes GPR78, IRE1α, TRAF2, JNK, and CHOP in the colon (*n* = 6). Data are shown as mean ± SEM. * *p* < 0.05, ** *p* < 0.01, *** *p* < 0.001 compared with HFHFr.

Loss of barrier integrity may promote intestinal inflammation [40]. We found that the mRNA levels of pro-inflammatory cytokines TNF-α, IL-1β, and IL-6 and chemokines CCL2 and CCL4 were significantly increased in the HFHFr group, but these inflammatory mediators were decreased in SFN groups (Figure 4A). Prior studies have largely focused on that, as LPS is a major pathogenic factor in the process of intestinal inflammation, it binds to TLR4 and activates the NF-κB pathway, causing an inflammatory response [28]. Intriguingly, the mRNA expressions of TLR4, MyD88, and NF-κB were significantly increased in the HFHFr group, and the intervention of SFN significantly reversed the elevation of these genes (Figure 4B). As demonstrated in Figure 4C–F, the intervention of SFN inhibited the activation of the TLR4/NF-κB signaling pathway in colon tissue. SFN may help to regulate inflammation and immune function by inhibiting the activation of the colonic TLR4/NF-κB pathway to reduce intestinal mucosal inflammation and colonic mucosal damage.

ERS in intestinal epithelial cells is associated with the activation of the host immune response and is the main factor in the pathogenesis of intestinal diseases [41]. We next investigated the effect of SFN on ERS-related marker genes in colon tissue. The mRNA expressions of GPR78 and IRE1α were increased in the HFHFr group, while the SFN intervention group significantly prevented the expression of ERS genes (Figure 4G). Additionally, we detected the downstream pathway genes of IRE1α and observed that the SFN group reduced the mRNA expression of tumor necrosis factor-receptor-associated factor 2 (TRAF2), c-Jun N-terminal kinase (JNK), and CHOP (Figure 4G). Taken together, the above results suggest that SFN intervention alleviates intestinal inflammation induced by the HFHFr diet.

### 3.5. SFN Improves Inflammation by Inhibiting the TLR4/NF-κB Signaling Pathway in the Liver of HFHFr Mice

**Figure 5:**
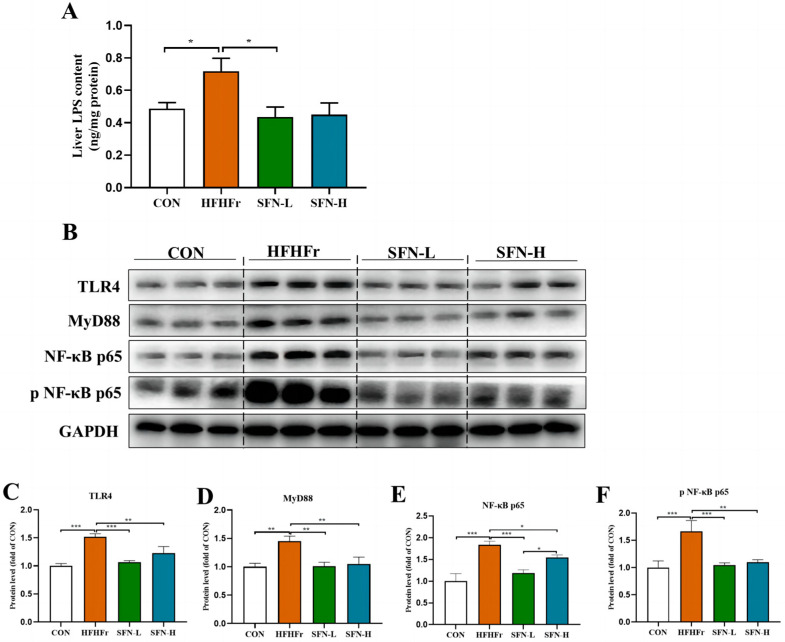
SFN improves hepatic inflammation by inhibiting the TLR4/MyD88/NF-κB signaling pathway in HFHFr mice. (**A**) Levels of LPS in the liver (*n* = 6). (**B**–**F**) The protein expression of TLR4, MyD88, NF–κB p65, and phosphorylation of NF–κB p65 in the liver were detected by Western blot (*n* = 3). Data are shown as mean ± SEM. * *p* < 0.05, ** *p* < 0.01, *** *p* < 0.001 compared with HFHFr.

Disrupted gut barriers lead to LPS leakage into the circulation through gut enterocytes towards the liver through the portal vein due to increased gut permeability. Hence, we detected the LPS levels in the mice livers by ELISA. As shown in Figure 5A, SFN treatment significantly reduced the LPS level in the liver. The ability to recognize the increased LPS, TLR4 and their downstream pathway has shown dominance in the mechanisms of the microbiota-gut-liver axis [42]. LPS is a key factor in inducing an inflammatory response in liver tissue and plays an important role in liver injury through the LPS-TLR4 signaling pathway [43]. Interestingly, SFN inhibited LPS-triggered TLR4/NF-κB signal pathway activation, as evidenced by decreased protein expressions of TLR4, MyD88, and NF-κB (Figure 5B–E). The activation of NF-κB is responsible for downstream inflammatory cytokines synthesis, and we further found that SFN inhibited phosphorylation of NF-κB (Figure 5B,F). As a downstream transcription factor of the TLR4 pathway, NF-κB is responsible for stimulating the production of major pro-inflammatory cytokines [44], and the generation of TNF-α, IL-1β, and IL-6 in the livers was confirmed in Figure 1G,H. Therefore, these results suggest that SFN intervention can improve the hepatic inflammation of NAFLD by reducing LPS translocation and inhibiting the activation of its downstream TLR4/NF-κB signaling pathway.

### 3.6. Correlation Supports That SFN Improves the Microbe-Gut-Liver Axis of HFHFr Mice

**Figure 6:**
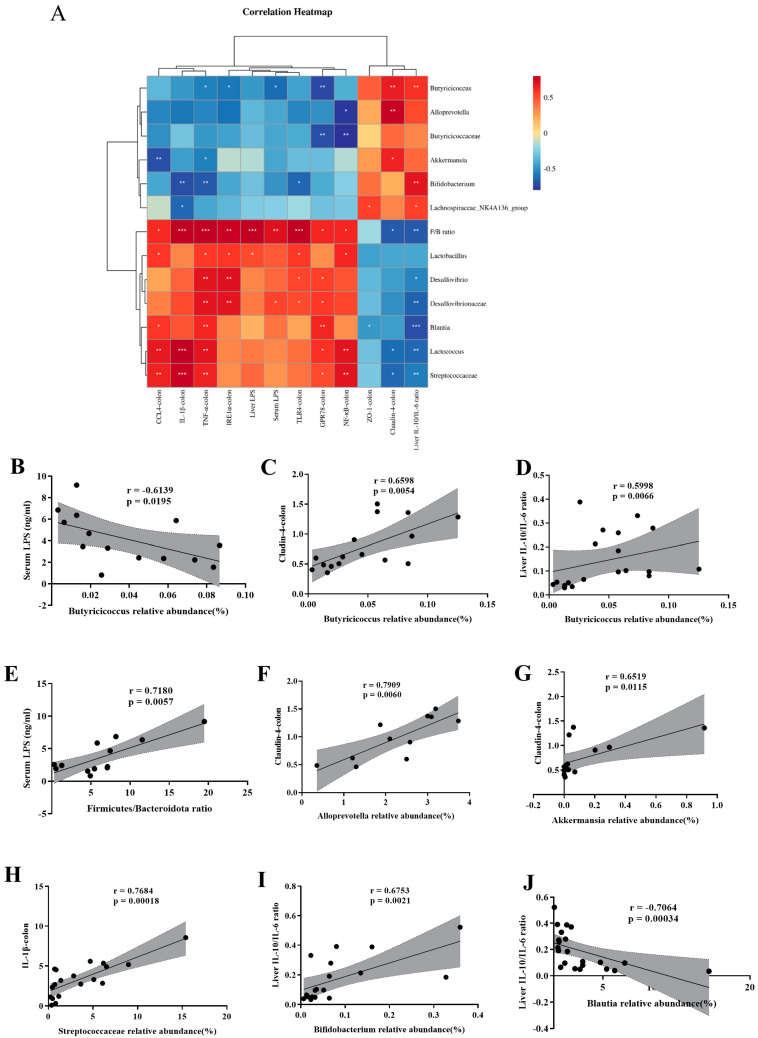
Spearman correlation analysis supports that SFN ameliorates HFHFr mice via the microbe–gut–liver axis. (**A**) Heatmap of the association between microbiota and some other experimental results. The intensity of the color is proportional to the intensity of the Spearman correlation. (**B**) Serum LPS levels were negatively correlated with the relative abundance of *Butyricicoccus*. (**C**) Relative mRNA expression of Claudin-4 in the colon was positively correlated with the relative abundance of *Butyricicoccus*. (**D**) IL-10/IL-6 ratio in the liver measured by ELISA was positively correlated with the relative abundance of *Butyricicoccus*. (**E**) Serum LPS levels were positively correlated with the Firmicutes/Bacteroidota ratio. (**F**) Relative mRNA expression of Claudin-4 in the colon was positively correlated with the relative abundance of *Alloprevotell*. (**G**) Relative mRNA expression of Claudin-4 in the colon was positively correlated with the relative abundance of *Akkermansia*. (**H**) Relative mRNA expression of IL-1β in the colon was positively correlated with the relative abundance of Streptococcaceae. (**I**) IL-10/IL-6 ratio in the liver measured by ELISA was positively correlated with the relative abundance of *Bifidobacterium*. (**J**) IL-10/IL-6 ratio in the liver measured by ELISA was negatively correlated with the relative abundance of *Blautia*. * *p* < 0.05, ** *p* < 0.01, *** *p* < 0.001 compared with HFHFr.

We performed a Spearman correlation heatmap to analyze the pathogenesis of the microbe–gut–liver axis in the HFHFr-induced NAFLD. Figure 6A shows the F/B ratio and the abundance of Streptococcaceae, *Lactococcus*, *Blautia*, Desulfovibrionaceae, *Desulfovibrio*, and *Lactobacillus* were positively related to colonic inflammatory genes, colonic endoplasmic reticulum stress genes, liver LPS levels, and serum LPS levels. Furthermore, the relative abundance of these gut bacteria was negatively correlated with the colonic tight junction protein genes ZO-1, Claudin-4, and the ratio of IL-10/IL-6 cytokines in the liver, while the relative abundances of *Lachnospiraceae_NK4A136_group*, *Bifidobacterium*, *Akkermansia*, *Allprevotella*, Butyricicoccaceae, and *Butyricicoccus* showed opposite trends to these experimental results. The results demonstrate that SFN intervention attenuates gut microbial dysregulation and protects mice from HFHFr diet-induced NAFLD.

Interestingly, we observed that the relative abundance of *Butyricicoccus* was significantly negatively correlated with the serum LPS levels (r = −0.6139, *p* = 0.0195) (Figure 6B) and was positively correlated with Claudin-4 (r = 0.6598, *p* = 0.0054) (Figure 6C) in the colon and the cytokine IL-10/IL-6 in the liver (r = 0.5998, *p* = 0.0066) (Figure 6D). These findings indicated that *Butyricicoccus* was the key bacterium most able to exert beneficial effects through the gut-liver axis after SFN intervention. Additionally, serum LPS levels positively correlated with the F/B ratio (r = 0.6484, *p* = 0.0196) (Figure 6E). Furthermore, the F/B ratio positively related to the immune-activated pathway genes, inflammatory genes, and endoplasmic reticulum stress marker genes in the colon, and was negatively correlated with the colon Claudin-4 and liver IL-10/IL-6 cytokines ratios (Figure 6A). These results showed that HFHFr diet-induced gut dysbiosis led to increased gut permeability and plasma LPS, thereby promoting low-grade inflammation [45]. The expression of Claudin-4 in the colon was positively correlated with the relative abundance of *Allprevotella* and *Akkermansia* (r = 0.7909, *p* = 0.0060; r = 0.6519, *p* = 0.0118) (Figure 6F–G). In addition, the expression of IL-1β in the colon was positively correlated with the relative abundance of Streptococcaceae (r = 0.7684, *p* = 0.00018) (Figure 6H). The liver cytokine IL-10/IL-6 ratio was negatively correlated with the relative abundance of *Blautia* (r = −0.7064, *p* = 0.00034) (Figure 6I) and positively correlated with the relative abundance of *Bifidobacterium* (r = 0.6753, *p* = 0.0021) (Figure 6J). Hence, we conclude that the microbe–gut–liver axis is involved in the development of HFHFr diet-induced NAFLD and can be improved by the SFN intervention.

## 4. Discussion

Studies have shown that HFHFr diets most closely recapitulate the human phenotype of NAFLD [46], and drinking water with excessive fructose can lead to barrier deterioration [47]. Therefore, we adopted an HFHFr diet to construct the NAFLD model in mice. Existing studies on SFN against NAFLD focused on inhibiting hepatic liposynthesis and steatosis [23,48], improving insulin resistance [21], antioxidant effects after NRF2 activation [49], and protecting mitochondrial functions [22]. Nevertheless, the protective effect of SFN against NAFLD in the gut–liver axis remains unknown. In this article, we primarily revealed that SFN protected against NAFLD by enhancing the gut barrier, modulating gut microbiota, and LPS-mediated gut–liver axis.

Studies on animals and humans have reported altered gut microbes in NAFLD individuals. Gut microbiota dysbiosis was observed in HFHFr-induced obese rats [42]. We observed that SFN decreased the F/B ratio and the abundance of *Lactobacillus*, *Lactococcus*, *Blautia*, *Dubosiella*, and *Alistipes* at the phylum level in HFHFr mice. These bacteria are genera associated with obesity-induced metabolic diseases, and their relative abundances significantly increased in the gut of mice following a high-fat diet or a high-fat and high-fructose diet [36,50,51,52]. Among them, the *Blautia* abundance positively correlated with fecal deoxycholic acid (DCA) levels in cirrhotic patients [50], and damage to the intestinal barrier [53]. *Desulfovibrio* is a sulfate-reducing bacterium that produces LPS, induces inflammation, and is associated with the development of metabolic syndrome [54]. Our findings suggested that SFN treatment significantly decreased the relative abundance of *Desulfovibrio*, indicating that SFN may modulate the gut microbiota in NAFLD mice by reducing harmful bacteria, inhibiting LPS production, and reducing inflammation.

More importantly, some beneficial bacterial genera (e.g., *Alloprevotella, Lachnospiraceae_NK4A136_group, Butyricicoccus, Bifidobacterium,* and *Akkermansia*) were increased by SFN in HFHFr-fed mice. *Alloprevotella* is a short-chain fatty acid-producing (SCFA) bacterium and improved NAFLD by dietary intervention in rodents [55]. The *Lachnospiraceae _NK4A136_ group* are butyrate-producing bacteria, which protect the intestinal mucosa and reduce host inflammation [56]. *Bifidobacterium* is an important health-promoting gut bacteria. It has been reported to improve liver inflammation in NAFLD and upregulate colon-tight junction genes to improve intestinal inflammation [57]. *Butyricicoccus*, a microorganism that primarily colonizes the associated surfaces of the colonic mucosa, contributes to the host’s metabolite butyrate production, which is essential for maintaining cellular homeostasis [58]. Consistent with existing studies on SFN improving *Butyricicoccus* in the gut microbiota in DSS-induced colitis [59], our results showed that SFN improved gut epithelium. The correlation analysis showed that *Butyricicoccus* was the key beneficial bacterium in the gut–liver axis regulated by the SFN intervention, and we hypothesized that its improvement potential relates to its butyrate-producing ability. Butyrates act as a colonic energy source for cells protecting the integrity of the intestinal epithelial barrier and therefore has anti-inflammatory effects. Subsequently, the beneficial role of *Butyricicoccus* in NAFLD deserves further exploration. Therefore, SFN may protect the gut microbiota of NAFLD mice induced by the HFHFr diet by increasing the abundance of beneficial genera.

Most of the existing studies on the protective effect of SFN on intestinal inflammation have focused on DSS-induced colitis. These studies claimed that SFN protects against colitis by reducing the expression of intestinal mucosal inflammatory biomarkers and increasing the expression of NRF2-dependent genes [60]. Additionally, SFN significantly ameliorated the BBN-induced increase in intestinal permeability and disruption of the intestinal barrier in bladder cancer by increasing tight junction protein expressions [61]. Impaired intestinal epithelial barrier function leads to intestinal inflammation and increased intestinal permeability [62]. For the first time, we figured out that the SFN intervention protects intestinal barrier functions in NAFLD mice. The expression of GPR78, an ERS marker, and pro-apoptotic proteins in the jejunum and ileum was increased by the HFD diet [63]. Previous studies have shown that IRE1α directly interacts with TRAF2 in the cytoplasm, thereby activating the JNK pathway, and integrating ER protein folding interference with inflammatory and apoptosis signaling pathways [64]. Our results showed that these ERS-related genes were upregulated in the colon of HFHFr mice and were decreased by SFN treatment, indicating that SFN protected intestinal barrier integrity by regulating ERS-related signal transduction.

In this study, we highlighted that the accumulation of gut-derived LPS in the blood may lead to liver injury and gut inflammation by triggering an inflammatory response. Existing studies have reported that abnormally translocated LPS from enteric pathogens binds to TLR4 and activates the transcription factor NF-κB, which is responsible for inflammatory cytokine synthesis, ultimately leading to liver injury and hepatic steatosis [65]. We found that TLR4 (a cell surface pattern recognition receptor) was significantly increased in the livers and colons of HFHFr-diet mice. The expression of TLR4 increased in hepatic steatosis individuals and is associated with raising serum LPS levels and intestinal barrier disruption [66], suggesting that the LPS/TLR4 pathway is one of the major mechanisms acting on the gut–liver axis. For example, luteolin inhibits the TLR4 signaling pathway in the liver, thereby reducing the secretion of pro-inflammatory factors and improving HFD-induced NAFLD [67]. Likewise, the anti-inflammatory activity of green tea extract against NAFLD is mediated in a TLR4-dependent manner and prevents NASH-induced inflammation by limiting gut-derived LPS translocation and TLR4 activation [68]. TLR4, MyD88, and NF-κB p65 protein levels were found to be elevated in the livers and colons of NAFLD mice and improved after SFN treatment. In conclusion, we observed that SFN dietary intervention reduced lipid accumulation and improved hepatic steatosis in the HFHFr mice. More precisely, it reduced inflammatory cytokines by inhibiting the hepatic TLR4/NF-κB pathway and increased the anti-inflammatory factors production.

Additionally, the main function of SFN in the body is that it is easy to interact with the thiol group of the protein to form a stable covalent bond, but this bond is not dose-dependent [69]. This may be an important reason why high-dose SFN did not improve hepatic and intestinal inflammation in the NAFLD model as well as low-dose SFN in this study.

## 5. Conclusions

**Figure 7:**
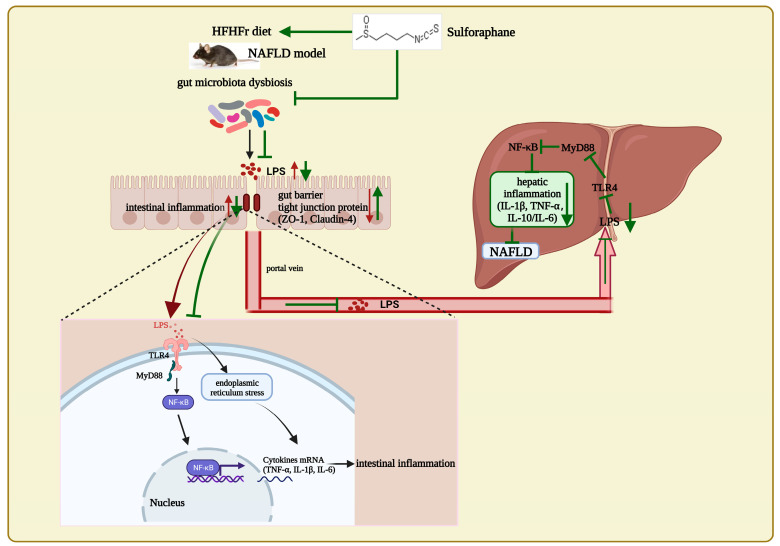
In mice with NAFLD induced by a high-fat, high-fructose diet, SFN supplement improves the compositions of gut microbes, increases the intestinal tight junction protein ZO-1, reduces serum LPS, and inhibits LPS/TLR4 and ERS pathway to reduce intestinal inflammation. Thus, SFN protects the intestinal integrity in NAFLD mice and declines the gut-derived LPS translocations to the liver. Moreover, SFN reduces the liver LPS level and inhibits the LPS/TLR4 pathway activations, thus inhibiting the pro-inflammatory cytokines and alleviating liver inflammation to improve NAFLD.

In conclusion, this study discovers that long-term SFN supplementation improves gut microbial composition in the HFHFr diet-induced NAFLD mice. Notably, dietary intervention with SFN reduces the F/B ratio; improved *Alloprevotella, Lachnospiraceae_NK4A136_group*, *Butyricicoccus*, and *Bifidobacterium* (important for maintaining gut barrier integrity); and inhibits the proliferation and growth of *Lactobacillus*, *Lactococcus*, *Blautia*, *Dubosiella*, and *Alistipes*. Additionally, SFN enhances the tight junction proteins in the colon, inhibits the LPS/TLR4 signaling pathway and endoplasmic reticulum stress in the intestine, and improves intestinal inflammation. As a consequence, it maintains the intestinal barrier integrity, reduces the translocation of intestinal-derived LPS, and inhibits the liver LPS/TLR4 signaling pathway to improve hepatic steatosis and steatohepatitis (Figure 7). Nonetheless, this study has the limitation of not validating the effect of SFN against NAFLD in vitro. In summary, we highlight that SFN prevents the occurrence of NAFLD by modulating the gut microbiota in mice and inhibiting the LPS-TLR4-NF-κB inflammatory pathway, implying that SFN is a potential natural product for the prevention and treatment of NAFLD.

## Data Availability

The data supporting the findings of this study are available from the corresponding author upon reasonable request.

## Supplementary Materials

The following supporting information can be downloaded at: https://www.mdpi.com/article/10.3390/nu15030743/s1, Table S1: title: Primer sequences used for RT-qPCR.

## Abbreviations

ALT, alanine aminotransferase; AST, aspartate aminotransferase; CCL2, chemokine (C-C motif) ligand 2; CCL4, chemokine (C-C motif) ligand 4; CHOP, C/EBP-homologous protein; ERS, endoplasmic reticulum stress; FGF, fibroblast growth factor; GPR78, G protein-coupled receptor 78; IL-1β, interleukin-1β; IL-6, interleukin-6; IL-10, interleukin-10; IRE1α, inositol-requiring enzyme-1α; JNK, c-Jun N-terminal kinase; LPS, lipopolysaccharide; MyD88, myeloid differentiation primary response 88; NAFLD, non-alcoholic fatty liver disease; NASH, non-alcoholic steatohepatitis; NF-κB, nuclear factor-κB; NRF2, nuclear factor erythroid 2-related factor 2; TLR4, Toll-like receptor 4; TNF-α, tumor necrosis factor-α; TRAF2, tumor necrosis factor-receptor-associated factor 2; TG, triglyceride; UPR, unfolded protein response; ZO-1, zonula occluden-1.

## References

[1] Corey K.E., Pitts R., Lai M., Loureiro J., Masia R., Osganian S.A., Gustafson J.L., Hutter M.M., Gee D.W., Meireles O.R. (2022). ADAMTSL2 protein and a soluble biomarker signature identify at-risk non-alcoholic steatohepatitis and fibrosis in adults with NAFLD. J. Hepatol..

[2] Porras D., Nistal E., Martínez-Flórez S., Pisonero-Vaquero S., Olcoz J.L., Jover R., González-Gallego J., García-Mediavilla M.V., Sánchez-Campos S. (2017). Protective effect of quercetin on high-fat diet-induced non-alcoholic fatty liver disease in mice is mediated by modulating intestinal microbiota imbalance and related gut-liver axis activation. Free. Radic. Biol. Med..

[3] Soderborg T.K., Clark S.E., Mulligan C.E., Janssen R.C., Babcock L., Ir D., Young B., Krebs N., Lemas D.J., Johnson L.K. (2018). The gut microbiota in infants of obese mothers increases inflammation and susceptibility to NAFLD. Nat. Commun..

[4] Albillos A., de Gottardi A., Rescigno M. (2020). The gut-liver axis in liver disease: Pathophysiological basis for therapy. J. Hepatol..

[5] Fan J., Sun J., Li T., Yan X., Jiang Y. (2022). Nuciferine prevents hepatic steatosis associated with improving intestinal mucosal integrity, mucus-related microbiota and inhibiting TLR4/MyD88/NF-κB pathway in high-fat induced rats. J. Funct. Foods.

[6] Zhang X., Coker O.O., Chu E.S., Fu K., Lau H.C.H., Wang Y.X., Chan A.W.H., Wei H., Yang X., Sung J.J.Y. (2021). Dietary cholesterol drives fatty liver-associated liver cancer by modulating gut microbiota and metabolites. Gut.

[7] Zhou D., Pan Q., Xin F.Z., Zhang R.N., He C.X., Chen G.Y., Liu C., Chen Y.W., Fan J.G. (2017). Sodium butyrate attenuates high-fat diet-induced steatohepatitis in mice by improving gut microbiota and gastrointestinal barrier. World J. Gastroenterol..

[8] Nighot M., Al-Sadi R., Guo S., Rawat M., Nighot P., Watterson M.D., Ma T.Y. (2017). Lipopolysaccharide-Induced Increase in Intestinal Epithelial Tight Permeability Is Mediated by Toll-Like Receptor 4/Myeloid Differentiation Primary Response 88 (MyD88) Activation of Myosin Light Chain Kinase Expression. Am. J. Pathol..

[9] Miele L., Valenza V., La Torre G., Montalto M., Cammarota G., Ricci R., Mascianà R., Forgione A., Gabrieli M.L., Perotti G. (2009). Increased intestinal permeability and tight junction alterations in nonalcoholic fatty liver disease. Hepatology.

[10] Peng J.H., Leng J., Tian H.J., Yang T., Fang Y., Feng Q., Zhao Y., Hu Y.Y. (2018). Geniposide and Chlorogenic Acid Combination Ameliorates Non-alcoholic Steatohepatitis Involving the Protection on the Gut Barrier Function in Mouse Induced by High-Fat Diet. Front. Pharmacol..

[11] Federico A., Dallio M., Godos J., Loguercio C., Salomone F. (2016). Targeting gut-liver axis for the treatment of nonalcoholic steatohepatitis: Translational and clinical evidence. Transl. Res..

[12] Mouries J., Brescia P., Silvestri A., Spadoni I., Sorribas M., Wiest R., Mileti E., Galbiati M., Invernizzi P., Adorini L. (2019). Microbiota-driven gut vascular barrier disruption is a prerequisite for non-alcoholic steatohepatitis development. J. Hepatol..

[13] Brooks-Worrell B.M., Palmer J.P. (2019). Setting the Stage for Islet Autoimmunity in Type 2 Diabetes: Obesity-Associated Chronic Systemic Inflammation and Endoplasmic Reticulum (ER) Stress. Diabetes Care.

[14] Zhao L., Liang J., Chen F., Tang X., Liao L., Liu Q., Luo J., Du Z., Li Z., Luo W. (2021). High carbohydrate diet induced endoplasmic reticulum stress and oxidative stress, promoted inflammation and apoptosis, impaired intestinal barrier of juvenile largemouth bass (*Micropterus salmoides*). Fish Shellfish Immunol..

[15] Carpino G., Del Ben M., Pastori D., Carnevale R., Baratta F., Overi D., Francis H., Cardinale V., Onori P., Safarikia S. (2020). Increased Liver Localization of Lipopolysaccharides in Human and Experimental NAFLD. Hepatology.

[16] Wang Q., Ou Y., Hu G., Wen C., Yue S., Chen C., Xu L., Xie J., Dai H., Xiao H. (2020). Naringenin attenuates non-alcoholic fatty liver disease by down-regulating the NLRP3/NF-κB pathway in mice. Br. J. Pharmacol..

[17] Zhang H., Gao X., Chen P., Wang H. (2022). Protective Effects of Tiaoganquzhi Decoction in Treating inflammatory Injury of Nonalcoholic Fatty liver Disease by Promoting CGI-58 and Inhibiting Expression of NLRP3 Inflammasome. Front. Pharmacol..

[18] Tsukamoto H., Takeuchi S., Kubota K., Kobayashi Y., Kozakai S., Ukai I., Shichiku A., Okubo M., Numasaki M., Kanemitsu Y. (2018). Lipopolysaccharide (LPS)-binding protein stimulates CD14-dependent Toll-like receptor 4 internalization and LPS-induced TBK1-IKKϵ-IRF3 axis activation. J. Biol. Chem..

[19] Friedman S.L., Neuschwander-Tetri B.A., Rinella M., Sanyal A.J. (2018). Mechanisms of NAFLD development and therapeutic strategies. Nat. Med..

[20] Youn H.S., Kim Y.S., Park Z.Y., Kim S.Y., Choi N.Y., Joung S.M., Seo J.A., Lim K.M., Kwak M.K., Hwang D.H. (2010). Sulforaphane suppresses oligomerization of TLR4 in a thiol-dependent manner. J. Immunol..

[21] Nagata N., Xu L., Kohno S., Ushida Y., Aoki Y., Umeda R., Fuke N., Zhuge F., Ni Y., Nagashimada M. (2017). Glucoraphanin Ameliorates Obesity and Insulin Resistance Through Adipose Tissue Browning and Reduction of Metabolic Endotoxemia in Mice. Diabetes.

[22] Xu L., Nagata N., Ota T. (2019). Impact of Glucoraphanin-Mediated Activation of Nrf2 on Non-Alcoholic Fatty Liver Disease with a Focus on Mitochondrial Dysfunction. Int. J. Mol. Sci..

[23] Wu Y.K., Ren Z.N., Zhu S.L., Wu Y.Z., Wang G., Zhang H., Chen W., He Z., Ye X.L., Zhai Q.X. (2021). Sulforaphane ameliorates non-alcoholic fatty liver disease in mice by promoting FGF21/FGFR1 signaling pathway. Acta Pharmacol. Sin..

[24] Wei L.Y., Zhang J.K., Zheng L., Chen Y. (2022). The functional role of sulforaphane in intestinal inflammation: A review. Food Funct..

[25] Tian S., Wang Y., Li X., Liu J., Wang J., Lu Y. (2021). Sulforaphane Regulates Glucose and Lipid Metabolisms in Obese Mice by Restraining JNK and Activating Insulin and FGF21 Signal Pathways. J. Agric. Food Chem..

[26] Xu X., Sun S., Liang L., Lou C., He Q., Ran M., Zhang L., Zhang J., Yan C., Yuan H. (2021). Role of the Aryl Hydrocarbon Receptor and Gut Microbiota-Derived Metabolites Indole-3-Acetic Acid in Sulforaphane Alleviates Hepatic Steatosis in Mice. Front. Nutr..

[27] Lee S., Kim J., Seo S.G., Choi B.R., Han J.S., Lee K.W., Kim J. (2014). Sulforaphane alleviates scopolamine-induced memory impairment in mice. Pharmacol. Res..

[28] Tian B., Zhao J., Zhang M., Chen Z., Ma Q., Liu H., Nie C., Zhang Z., An W., Li J. (2021). *Lycium ruthenicum* Anthocyanins Attenuate High-Fat Diet-Induced Colonic Barrier Dysfunction and Inflammation in Mice by Modulating the Gut Microbiota, Molecular nutrition. Food Res..

[29] Yan S., Shi R., Li L., Ma S., Zhang H., Ye J., Wang J., Pan J., Wang Q., Jin X. (2019). Mannan Oligosaccharide Suppresses Lipid Accumulation and Appetite in Western-Diet-Induced Obese Mice Via Reshaping Gut Microbiome and Enhancing Short-Chain Fatty Acids Production. Mol. Nutr. Food Res..

[30] Guo M., Huang K., Chen S., Qi X., He X., Cheng W.H., Luo Y., Xia K., Xu W. (2014). Combination of metagenomics and culture-based methods to study the interaction between ochratoxin a and gut microbiota. Toxicol. Sci..

[31] Chen S.L., Li J.P., Li L.F., Zeng T., He X. (2016). Elevated Preoperative Serum Alanine Aminotransferase/Aspartate Aminotransferase (ALT/AST) Ratio Is Associated with Better Prognosis in Patients Undergoing Curative Treatment for Gastric Adenocarcinoma. Int. J. Mol. Sci..

[32] Safari Z., Gérard P. (2019). The links between the gut microbiome and non-alcoholic fatty liver disease (NAFLD). Cell. Mol. Life Sci. CMLS.

[33] Leung C., Rivera L., Furness J.B., Angus P.W. (2016). The role of the gut microbiota in NAFLD. Nat. Rev. Gastroenterol. Hepatol..

[34] Bhatt B., Zeng P., Zhu H., Sivaprakasam S., Li S., Xiao H., Dong L., Shiao P., Kolhe R., Patel N. (2018). Gpr109a Limits Microbiota-Induced IL-23 Production To Constrain ILC3-Mediated Colonic Inflammation. J. Immunol..

[35] Wang Y., Yao W., Li B., Qian S., Wei B., Gong S., Wang J., Liu M., Wei M. (2020). Nuciferine modulates the gut microbiota and prevents obesity in high-fat diet-fed rats. Exp. Mol. Med..

[36] Chen G., Chen D., Zhou W., Peng Y., Chen C., Shen W., Zeng X., Yuan Q. (2021). Improvement of Metabolic Syndrome in High-Fat Diet-Induced Mice by Yeast β-Glucan Is Linked to Inhibited Proliferation of Lactobacillus and Lactococcus in Gut Microbiota. J. Agric. Food Chem..

[37] Moorthy M., Wie C.C., Mariño E., Palanisamy U.D. (2022). The Prebiotic Potential of Geraniin and Geraniin-Enriched Extract against High-Fat-Diet-Induced Metabolic Syndrome in Sprague Dawley Rats. Antioxidants.

[38] Li R., Yao Y., Gao P., Bu S. (2020). The Therapeutic Efficacy of Curcumin vs. Metformin in Modulating the Gut Microbiota in NAFLD Rats: A Comparative Study. Front. Microbiol..

[39] Krumbeck J.A., Rasmussen H.E., Hutkins R.W., Clarke J., Shawron K., Keshavarzian A., Walter J. (2018). Probiotic Bifidobacterium strains and galactooligosaccharides improve intestinal barrier function in obese adults but show no synergism when used together as synbiotics. Microbiome.

[40] Wang H., Wang G., Banerjee N., Liang Y., Du X., Boor P.J., Hoffman K.L., Khan M.F. (2021). Aberrant Gut Microbiome Contributes to Intestinal Oxidative Stress, Barrier Dysfunction, Inflammation and Systemic Autoimmune Responses in MRL/lpr Mice. Front. Immunol..

[41] Ma X., Dai Z., Sun K., Zhang Y., Chen J., Yang Y., Tso P., Wu G., Wu Z. (2017). Intestinal Epithelial Cell Endoplasmic Reticulum Stress and Inflammatory Bowel Disease Pathogenesis: An Update Review. Front. Immunol..

[42] Li K.P., Yuan M., Wu Y.L., Pineda M., Zhang C.M., Chen Y.F., Chen Z.Q., Rong X.L., Turnbull J.E., Guo J. (2022). A High-Fat High-Fructose Diet Dysregulates the Homeostatic Crosstalk Between Gut Microbiome, Metabolome, and Immunity in an Experimental Model of Obesity. Mol. Nutr. Food Res..

[43] An L., Wirth U., Koch D., Schirren M., Drefs M., Koliogiannis D., Nieß H., Andrassy J., Guba M., Bazhin A.V. (2022). The Role of Gut-Derived Lipopolysaccharides and the Intestinal Barrier in Fatty Liver Diseases. J. Gastrointest. Surg..

[44] Hu Q., Zhang W., Wu Z., Tian X., Xiang J., Li L., Li Z., Peng X., Wei S., Ma X. (2021). Baicalin and the liver-gut system: Pharmacological bases explaining its therapeutic effects. Pharmacol. Res..

[45] Panasevich M.R., Meers G.M., Linden M.A., Booth F.W., Perfield J.W., Fritsche K.L., Wankhade U.D., Chintapalli S.V., Shankar K., Ibdah J.A. (2018). High-fat, high-fructose, high-cholesterol feeding causes severe NASH and cecal microbiota dysbiosis in juvenile Ossabaw swine. Am. J. Physiol. Endocrinol. Metab..

[46] Im Y.R., Hunter H., de Gracia Hahn D., Duret A., Cheah Q., Dong J., Fairey M., Hjalmarsson C., Li A., Lim H.K. (2021). A Systematic Review of Animal Models of NAFLD Finds High-Fat, High-Fructose Diets Most Closely Resemble Human NAFLD. Hepatology.

[47] Todoric J., Di Caro G., Reibe S., Henstridge D.C., Green C.R., Vrbanac A., Ceteci F., Conche C., McNulty R., Shalapour S. (2020). Fructose stimulated de novo lipogenesis is promoted by inflammation. Nat. Metab..

[48] Li J., Xie S., Teng W. (2021). Sulforaphane Attenuates Nonalcoholic Fatty Liver Disease by Inhibiting Hepatic Steatosis and Apoptosis. Nutrients.

[49] Yu H., Jiang X., Dong F., Zhang F., Ji X., Xue M., Yang F., Chen J., Hu X., Bao Z. (2021). Lipid accumulation-induced hepatocyte senescence regulates the activation of hepatic stellate cells through the Nrf2-antioxidant response element pathway. Exp. Cell Res..

[50] Lin H., An Y., Tang H., Wang Y. (2019). Alterations of Bile Acids and Gut Microbiota in Obesity Induced by High Fat Diet in Rat Model. J. Agric. Food Chem..

[51] Chen M., Zheng J., Zou X., Ye C., Xia H., Yang M., Gao Q., Yang Q., Liu H. (2021). *Ligustrum robustum* (Roxb.) blume extract modulates gut microbiota and prevents metabolic syndrome in high-fat diet-fed mice. J. Ethnopharmacol..

[52] Qiao Y., Zhang Z., Zhai Y., Yan X., Zhou W., Liu H., Guan L., Peng L. (2021). Apigenin Alleviates Obesity-Associated Metabolic Syndrome by Regulating the Composition of the Gut Microbiome. Front. Microbiol..

[53] Stenman L.K., Holma R., Eggert A., Korpela R. (2013). A novel mechanism for gut barrier dysfunction by dietary fat: Epithelial disruption by hydrophobic bile acids. Am. J. Physiol. Gastrointest. Liver Physiol..

[54] Xu P., Hong F., Wang J., Cong Y., Dai S., Wang S., Wang J., Jin X., Wang F., Liu J. (2017). Microbiome Remodeling via the Montmorillonite Adsorption-Excretion Axis Prevents Obesity-related Metabolic Disorders. eBioMedicine.

[55] Guo W.L., Pan Y.Y., Li L., Li T.T., Liu B., Lv X.C. (2018). Ethanol extract of *Ganoderma lucidum* ameliorates lipid metabolic disorders and modulates the gut microbiota composition in high-fat diet fed rats. Food Funct..

[56] Li D.P., Cui M., Tan F., Liu X.Y., Yao P. (2021). High Red Meat Intake Exacerbates Dextran Sulfate-Induced Colitis by Altering Gut Microbiota in Mice. Front. Nutr..

[57] Do M.H., Oh M.J., Lee H.B., Kang C.H., Yoo G., Park H.Y. (2022). *Bifidobacterium animalis* ssp. lactis MG741 Reduces Body Weight and Ameliorates Nonalcoholic Fatty Liver Disease via Improving the Gut Permeability and Amelioration of Inflammatory Cytokines. Nutrients.

[58] Devriese S., Eeckhaut V., Geirnaert A., Van den Bossche L., Hindryckx P., Van de Wiele T., Van Immerseel F., Ducatelle R., De Vos M., Laukens D. (2017). Reduced Mucosa-associated Butyricicoccus Activity in Patients with Ulcerative Colitis Correlates with Aberrant Claudin-1 Expression. J. Crohn’s Colitis.

[59] Zhang Y., Tan L., Li C., Wu H., Ran D., Zhang Z. (2020). Sulforaphane alter the microbiota and mitigate colitis severity on mice ulcerative colitis induced by DSS. AMB Express.

[60] Wagner A.E., Will O., Sturm C., Lipinski S., Rosenstiel P., Rimbach G. (2013). DSS-induced acute colitis in C57BL/6 mice is mitigated by sulforaphane pre-treatment. J. Nutr. Biochem..

[61] He C., Huang L., Lei P., Liu X., Li B., Shan Y. (2018). Sulforaphane Normalizes Intestinal Flora and Enhances Gut Barrier in Mice with BBN-Induced Bladder Cancer. Mol. Nutr. Food Res..

[62] Xu Q., Zhang R., Mu Y., Song Y., Hao N., Wei Y., Wang Q., Mackay C.R. (2022). Propionate Ameliorates Alcohol-Induced Liver Injury in Mice via the Gut-Liver Axis: Focus on the Improvement of Intestinal Permeability. J. Agric. Food Chem..

[63] Chen J., Yang Y., Yang Y., Dai Z., Kim I.H., Wu G., Wu Z. (2021). Dietary Supplementation with Glycine Enhances Intestinal Mucosal Integrity and Ameliorates Inflammation in C57BL/6J Mice with High-Fat Diet-Induced Obesity. J. Nutr..

[64] Hetz C. (2012). The unfolded protein response: Controlling cell fate decisions under ER stress and beyond. Nat. Rev. Mol. Cell Biol..

[65] Loomba R., Seguritan V., Li W., Long T., Klitgord N., Bhatt A., Dulai P.S., Caussy C., Bettencourt R., Highlander S.K. (2019). Gut Microbiome-Based Metagenomic Signature for Non-invasive Detection of Advanced Fibrosis in Human Nonalcoholic Fatty Liver Disease. Cell Metab..

[66] Cheng C., Tan J., Qian W., Zhang L., Hou X. (2018). Gut inflammation exacerbates hepatic injury in the high-fat diet induced NAFLD mouse: Attention to the gut-vascular barrier dysfunction. Life Sci..

[67] Liu X., Sun R., Li Z., Xiao R., Lv P., Sun X., Olson M.A., Gong Y. (2021). Luteolin alleviates non-alcoholic fatty liver disease in rats via restoration of intestinal mucosal barrier damage and microbiota imbalance involving in gut-liver axis. Arch. Biochem. Biophys..

[68] Li J., Sasaki G.Y., Dey P., Chitchumroonchokchai C., Labyk A.N., McDonald J.D., Kim J.B., Bruno R.S. (2018). Green tea extract protects against hepatic NFκB activation along the gut-liver axis in diet-induced obese mice with nonalcoholic steatohepatitis by reducing endotoxin and TLR4/MyD88 signaling. J. Nutr. Biochem..

[69] Ji Y., Kuo Y., Morris M.E. (2005). Pharmacokinetics of dietary phenethyl isothiocyanate in rats. Pharm. Res..

