# Supplementary material for: Sulforaphane Ameliorates Nonalcoholic Fatty Liver Disease Induced by High-Fat and High-Fructose Diet via LPS/TLR4 in the Gut–Liver Axis
Source: Nutrients. 2023 Feb 1;15(3):743. doi: 10.3390/nu15030743 (PMC9920698; doi:10.3390/nu15030743)
Supplement: Supplementary file 1 [file nutrients-15-00743-s001.zip › nutrients-2159986-supplementary.pdf]

Supplementary material

Table S1 Primer sequences used for RT-qPCR

Gene	Forward primer (5'→ 3')	Reverse primer (5'→ 3')
β-actin	GGCTGTATTCCCCTCCATCG	CCAGTTGGTAACAATGCCATGT
ZO-1	ACCACCAACCCGAGAAGAC	CAGGAGTCATGGACGCACA
Claudin-4	GTCCTGGGAATCTCCTTGGC	TCTGTGCCGTGACGATGTTG
TLR4	ATGGCATGGCTTACACCACC	GAGGCCAATTTTGTCTCCACA
MyD88	TCATGTTCTCCATACCCTTGGT	AAACTGCGAGTGGGGTCAG
NF-κB	ATGGCAGACGATGATCCCTAC	TGTTGACAGTGGTATTTCTGGTG
TNF-α	GACGTGGAACTGGCAGAAGAG	TTGGTGGTTTGTGAGTGTGAG
IL-1β	GCAACTGTTCCTGAACTCAACT	ATCTTTTGGGGTCCGTCAACT
IL-6	TGAACAACGATGATGCACTTG	CTGAAGGACTCTGGCTTTGTC
CCL2	TTAAAAACCTGGATCGGAACCAA	GCATTAGCTTCAGATTTACGGGT
CCL4	TTCCTGCTGTTTCTCTTACACCT	CTGTCTGCCTCTTTTGGTCAG
GPR78	ACTTGGGGACCACCTATTCCT	ATCGCCAATCAGACGCTCC
IRE1α	ACACTGCCTGAGACCTTGTTG	GGAGCCCGTCCTCTTGCTA
TRAF2	AGAGAGTAGTTCGGCCTTTCC	GTGCATCCATCATTGGGACAG
JNK	AGCAGAAGCAAACGTGACAAC	GCTGCACACACTATTCCTTGAG
CHOP	CCACCACACCTGAAAGCAGAA	AGGTGAAAGGCAGGGACTCA
